# Autoimmune phenomena and spontaneous tumour regression. The role of carbonic anhydrase I

**DOI:** 10.1111/jcmm.16525

**Published:** 2021-04-04

**Authors:** Lenka Minichová, Ľudovít Škultéty, Ján Lakota

**Affiliations:** ^1^ Biomedical Center SAS Bratislava Slovakia; ^2^ Institute of Microbiology CAS Praha Slovakia; ^3^ Center of Experimental Medicine SAS Bratislava Slovakia; ^4^ Faculty of Management Comenius University Bratislava Slovakia

Dear Editor,

We have observed the spontaneous tumour regression in four patients (breast cancer, Hodgkin's disease, non‐Hodgkin's lymphoma and Ewing's sarcoma) who had relapsed/progressed after high‐dose chemotherapy (HDC) and autologous haematopoietic stem cell transplantation (ASCT).^1^ Patients' blood counts strongly resembled the blood counts in aplastic anaemia (aplastic anaemia (AA)‐like syndrome). Moreover, some of these patients' morphology of the bone marrow trephine biopsies was identical to the AA picture. It should be noted that in some patients who were treated by conventional chemotherapy only, a similar phenomenon has been observed, too (Lakota J, unpublished observation). In a provoking paper,^2^ the authors proposed that the autoimmune (anti‐tumour) activity against present malignancy also operates against haematopoietic stem cells. The final clinical and laboratory pattern in these patients is present as acquired aplastic anaemia (AA). This pathophysiological mechanism could explain pancytopenia, which frequently occurs in patients with haematological as well as non‐haematological malignancies. The conclusion would be that pancytopenia which is present in AA or AA‐like syndrome reflects an ongoing immune reaction against underlying malignancy.^2^ By analysis of the sera of patients with spontaneous tumour regression and (AA)‐like syndrome, we have shown that they contain antibodies against carbonic anhydrase I.^3^ These antibodies were polyclonal. Mapping of carbonic anhydrase isoform I (CA I), we detected four linear epitopes of CA I enzyme (DGLAV, NVGHS, SLKPI and SSEQL).^4^ We have analysed the sera of bona fide AA patients too. Anti‐CA I (auto)antibodies were detected in 38% of patients. Their presence was associated with poor response to ATG treatment (complete response 14%), short long‐term survival (36% at ten years) in contrast to antibody‐negative patients (complete response 64% and ten‐year survival 64%, overall *p*‐value = 0.003).^5^ After the epitope mapping, the anti‐CA I antibodies in this group of patients (bona fide AA) recognized the same linear candidates CA I epitopes—DGLAV, NVGHS, SLKPI and SSEQL.^6^ Antibodies against CA I have been presented in some autoimmune diseases, for example, systemic lupus erythematosus, Sjögren's syndrome, autoimmune/idiopathic chronic pancreatitis, connective tissue diseases and other rheumatic diseases (reviewed previously in Ref. [6]).

Crohn's disease and ulcerative colitis ((autoimmune) inflammatory bowel diseases (IBD)) are characterized by an inflammation of the large and/or small intestine. In 2012, the group from Ehime University (in a murine model) identified CA I as a dominant protein of faecal extracts (called caecal bacterial Ag (CBA)) which was related to the pathogenesis of IBD. Furthermore, the researchers established a therapeutic IBD approach where they used Ag‐pulsed regulatory dendritic cells (Reg‐DCs).^7^ First, they induced colitis by transfer of CD4+ CD25^‐^ T cells which were obtained from BALB/c mice and were transferred into SCID mice. Ag (ie CA I)‐pulsed Reg‐DCs_CAI_ induced the differentiation of regulatory T cells and blocked the progression of the colitis. The authors concluded that Reg‐DCs_CBA_ inhibited the progression of colitis which has been induced by CD4+ CD25^‐^ T cells and that CA I, a central antigen of CBA, had an essential role in suppressing the development of colitis.^7^ In a more recent paper, the group from Ehime University has shown that oral administration of CA I was able to induce antigen‐specific immune tolerance and generated Foxp3+ CD4+ CD25+ Tregs cells. This treatment protects from intestinal inflammation in a murine model.^8^ (The mechanism of oral tolerance is defined as the inhibition of the immune response to a specific antigen which can be induced in an antigen‐specific manner. In general, these effects are not limited to the local immunity only ie within the gut. Oral tolerance ameliorates autoimmune diseases, such as diabetes, multiple sclerosis, rheumatoid arthritis and uveitis. It does not have significant side effects).^9^ Finally, Yagi et al^10^ clearly showed that the T‐cell CA I epitope peptide interacts with MHC class II molecules that CA I 58‐73 peptide‐pulsed Reg‐DCs protects mice with experimental colitis, and that Reg‐DCs_CA__I 58‐73_ induces Ag‐specific Tregs.

Interestingly, the murine CA I 58‐73 fragment (KEIVNVGHSFHVIFDD) shows high sequence similarity (81.3%) with human CA I 58‐73 fragment (KEIINVGHSFHVNFED). As it has been previously shown,^6^ there has been an epitope overlap of the antibodies in (AA)‐like anaemia syndrome and the antibodies in bona fide AA (ie DGLAV, NVGHS, SLKPI and SSEQL). Analogous (albeit one) epitope overlap exists in the murine CA I 58‐73 fragment, which is 100% homologous with the human epitope—NVGHS. The murine CA I 58‐73 fragment protects mice with experimental colitis in a murine model for autoimmune IBD. On the other hand, the human fragment (NVGHS) acts as the target epitope of the antibodies against carbonic anhydrase I in patients with bona fide AA and (AA)‐like syndrome. Aplastic anaemia (AA)‐like syndrome has been observed only in patients who relapsed/progressed after HDC and ASCT and achieved spontaneous tumour regression (ie without any further treatment). The epitope ‘alignment’ in these cases is not only surprising, but it is also challenging. Will we go further, prepare monoclonal therapeutic antibodies, and possibly will be able to treat a wide variety of malignant tumours?

## CONFLICT OF INTEREST

The authors confirm that there is no conflict of interest.

## AUTHOR CONTRIBUTIONS

**Lenka Minichova:** Data curation (equal); Formal analysis (equal); Project administration (equal). **Ludovit Skultety:** Data curation (equal); Formal analysis (equal); Project administration (equal). **Jan Lakota:** Conceptualization (lead); Data curation (equal); Formal analysis (equal); Funding acquisition (lead); Project administration (equal).
